# The Role of Inflammation and Therapeutic Concepts in Diabetic Retinopathy—A Short Review

**DOI:** 10.3390/ijms24021024

**Published:** 2023-01-05

**Authors:** Krzysztof Gomułka, Michał Ruta

**Affiliations:** 1 Clinical Department of Internal Medicine, Pneumology and Allergology, Wroclaw Medical University, ul. M. Curie-Skłodowskiej 66, 50-369 Wrocław, Poland; 2 Clinical Department of Ophthalmology, 4th Military Clinical Hospital with Polyclinic, ul. Rudolfa Weigla 5, 50-981 Wrocław, Poland

**Keywords:** diabetes, inflammation, retinopathy, vascular endothelial growth factor, corticosteroid

## Abstract

Diabetic retinopathy (DR) as a microangiopathy is the most common complication in patients with diabetes mellitus (DM) and remains the leading cause of blindness among adult population. DM in its complicated pathomechanism relates to chronic hyperglycemia, hypoinsulinemia, dyslipidemia and hypertension—all these components in molecular pathways maintain oxidative stress, formation of advanced glycation end-products, microvascular changes, inflammation, and retinal neurodegeneration as one of the key players in diabetes-associated retinal perturbations. In this current review, we discuss the natural history of DR with special emphasis on ongoing inflammation and the key role of vascular endothelial growth factor (VEGF). Additionally, we provide an overview of the principles of diabetic retinopathy treatments, i.e., in laser therapy, anti-VEGF and steroid options.

## 1. Introduction

Diabetes mellitus (DM) is a group of metabolic disorders characterized by a high blood glucose level (hyperglycemia) over a prolonged period [1,2]. Classification of diabetes in clinical work includes a division into type 1 diabetes and type 2 diabetes. It varies in degree of heterogeneity in clinical manifestations, accurate diagnosis, comorbidities, and treatment [3,4,5]. Diabetes can cause many health complications both acute (e.g., diabetic ketoacidosis, hyperosmolar hyperglycemic state) and long-term (e.g., cardiovascular disease, stroke, chronic kidney disease, foot ulcers, and damage to the nerves or to the eyes) [6,7,8]. Diabetic retinopathy (DR) is a major microvascular complication of diabetes mellitus and remains the leading cause of visual loss in the adult population. It is estimated that diabetes might affect up to 34% of the worldwide population aged 40 and older by 2035 [9,10]—according to this data, DR is growing into a worldwide health problem as well. A study by Lin et al. [11] revealed that women with DM type 2 had a higher prevalence of diabetic retinopathy than men, but men suffered from more severe retinopathy, poor vision, or blindness. DR impacts not only the quality of life, but also predicted vascular and non-cancer mortality, prolonged QT interval, or life-threatening arrhythmia [12,13]. Considering clinical manifestations of vascular abnormalities in the retina, DR is divided into two stages: non-proliferative diabetic retinopathy (NPDR) and proliferative diabetic retinopathy (PDR). In the initial stage of NPDR, hyperglycemia and altered metabolic pathways lead to oxidative stress, leakage of multiple inflammatory cytokines and plasma proteins and then to the development of neurodegeneration, disruption of the blood-retinal barrier (BRB) and progressive retinal pathologies. Early hallmarks of NPDR, detected under fundus photography include increased vascular permeability, capillary occlusion, microaneurysms, dot intraretinal hemorrhage and hard exudates. As disease progresses, a more advanced stage of DR turns uncontrollably into PDR where severe hypoxia leads to neovascularization, vitreous hemorrhage, and retinal detachment such as traction retinal detachments (TRDs) and combined traction/rhegmatogenous retinal detachments (TRD/RRDs) which remains the most common reason for vitrectomy in patients with proliferative diabetic retinopathy [14,15]. There are four stages of diabetic retinopathy [16,17,18]:

1. Mild non-proliferative diabetic retinopathy (NPDR)—there may be no symptoms in this stage; microaneurysms develop on the tiny vessels in the retina, the light-sensitive back layer of the eyeball; leak fluid into the retina might be present.
2. Moderate NPDR—more vessels become weak and blocked; they begin to be swollen and distorted in size and lose their ability to properly transport blood.
3. Severe NPDR—more blood vessels become blocked which disrupts blood supply to areas in the retina with compensation by signaling the retina to grow up new blood vessels.
4. Proliferative diabetic retinopathy (PDR)—the most advanced stage of retinopathy where new, weak, and inefficient blood vessels grow along the inside surface of the retina and into the vitreous gel; they are more likely to leak and bleed causing retinal detachment.

The classification of DR according to ophthalmoscopic features is shown in Table 1.

The distortion of visual images, decrease in visual acuity and vision loss in patients with DM can occur at any stage of DR. The most common cause of vision loss in patients with DR is diabetic macular edema (DME) which is the result of swelling or thickening of the macula due to sub- and intra-retinal accumulation of fluid in the macula triggered by the breakdown of the BRB. Currently the mainstay of therapy for DR aim at managing the microvascular complications, including intravitreal administration of pharmacological agents with steroids as one possible option, along with laser photocoagulation and vitreoretinal surgery [11,17,19]. Here, we present a brief overview of insights into the pathophysiology of DR and chosen therapeutic targets and potential pharmacological agents being used in patients with DR. The present review aims to summarize the current knowledge concerning the inflammatory processes playing a crucial role in the development of DR, with special emphasis on vascular endothelial growth factor (VEGF) and focused on various aspects of treatment of DR. Its contribution is related to the previous research conducted on VEGF in various diseases and the treatment of patients with diabetic retinopathy using various methods by the authors.

## 2. Pathology in DR

Hyperglycemia is considered as an important factor in the pathogenesis of retinal microvascular damage which leads to DR. Metabolic pathways that are considered during hyperglycemia-induced vascular damage including advanced glycation end products (AGEs) accumulation, the protein kinase C (PKC) pathway, and the polyol and the hexosamine pathway. Additionally, dilation of blood vessels and blood flow changes are the earliest responses to hyperglycemia from retinal blood vessels in diabetic patients. Other hallmarks of the early events of DR are pericytes loss triggered by high glucose concentration with the following outpouching of capillary walls and microaneurysm formation, apoptosis of endothelial cells, and thickening of the basement membrane. Next, pronounced loss of pericytes and endothelial cells collectively contributes to the impairment of the BRB and results in capillary occlusion and ischemia. Retinal ischemia/hypoxia leads to activation of hypoxia-inducible factor 1 (HIF-1) and upregulation of angiogenic factors such as angiopoietins (Ang-1, Ang-2) and most vascular endothelial growth factor (VEGF) [9,20].

### 2.1. DR—An Inflammatory Disease

Originally, DR was considered a purely microvascular disease. Currently, chronic, low-grade inflammation plays a key role in the pathogenesis of DR that leads to changes in the retinal microcirculation and was detected widely in different stages of DR in both diabetic animal models and in the retinas of diabetic patients. This pathology affects neuronal and vascular components of the retina and what is more, it shows some similarities with chronic inflammatory diseases like infiltration of inflammatory cells, expression of different effectors such as cytokines responsible for damage to the retina, edema, neovascularization, or destruction of tissues.

#### 2.1.1. Role of Inflammatory Cells in DR

Leukostasis is an occlusion of retinal microvasculature by monocytes, macrophages and granulocytes and was reported in an animal model of DM and in the early stage of DR in patients [21,22,23]. Additionally, increased leukostasis might be correlated with endothelium damage and BRB impairment through the Fas (CD95)/Fas-ligand pathway. Leukocyte-endothelium adhesion occurs according to upregulation of leukocyte b2-integrins (CD11a, CD11b, CD18) and expression of endothelial cell adhesion molecules such as intercellular adhesion molecule-1 (ICAM-1), vascular cell adhesion molecule-1 (VCAM-1) and selectins (E-selectin). Additionally, the plasma expression of VCAM-1 and E-selectin is correlated with the severity of DR. Retinal glial cells, consisted of astrocytes, Müller cells and microglia, are responsible for structural support and maintaining homeostasis in the retina. Nevertheless, in the condition of hyperglycemia and oxidative stress, glial cells are dysfunctional and they enhance the production of proinflammatory cytokines (TNF-α, growth factors, IL-1β, IL-6) which are involved in the onset and amplification of inflammation in the diabetic retina. In addition, the secretion of proinflammatory cytokines by glial cells plays a role in the infiltration of monocytes and T lymphocytes and on the other hand chronic inflammation induces fibrotic processes which induce scar formation and then retinal detachment [24,25].

#### 2.1.2. Role of Inflammatory Chemokines in DR

Some chemokines have been shown to be involved in the pathogenesis of DR. In some recent studies, monocyte chemotactic protein-1 (MCP-1) and macrophage inflammatory protein-1 alpha (MIP-1a) have been reported to be elevated in diabetic patients [14,26,27]. Moreover, a level of other inflammatory cytokines such as interleukin 1 (IL-1), IL-6, IL-8, and tumor necrosis factor alpha (TNF-α) was upregulated in diabetic patients with DR. Additionally, there is a correlation between the presence of high levels of growth factors and inflammatory cytokines in the eye fluids and inflammation in patients with DR. In the retina, under hyperglycemic stress, microglia is activated, which leads to the upregulation of the Nuclear Factor kappa-light-chain-enhancer of activated B cells (NF-κB) followed by increase in oxidative stress with induction of pro-inflammatory cytokines, such as IL-1b, VEGF and TNF-α, chemokines, and adhesion molecules (E-selectin, ICAM-1). Above-described activation of the inflammatory process in DR causes the increase in vascular permeability, loss of pericytes, and the appearance of microaneurysms. Because the retina uses high quantities of glucose and oxygen to generate energy by using the mitochondrial electron transport chain (ETC), reactive oxygen species (ROS) and free electrons are increased during inflammatory process in DR which causes release into the cytosol harmful lipids, proteins and oxidized mitochondrial DNA (mtDNA). They are recognized as damage-associated molecular profiles (DAMP) by the Toll-like receptors TLR4, TLR9 and NLRP3, which in turn enhances the production and activation of pro-IL-1β and pro-caspase-1 [26,28].

### 2.2. VEGF

“Vascular Permeability Factor” (VPF) was described by Senger et al. in 1983 [29], then Ferrara and Henzel in 1989 [30] discovered its mitotic effect on endothelial cells and proposed the name vascular endothelial growth factor (VEGF). Nowadays, “a family” of vascular endothelial growth factors consists of several members: VEGF-A (called generally VEGF, the prototype molecule of a family, discovered first); VEGF-B, VEGF-C, and VEGF-D (also known as c-Fos-induced growth factor, FIGF); placenta growth factor (PlGF); and the viral VEGF-E encoded by strains D1701, NZ2 and NZ7 of the parapoxvirus Orf (which causes pustular dermatitis) (Table 2). VEGF itself is a heparin-binding, homodimer glycoprotein; its weight is 46 kDa, with a different number of amino-acids that are produced in human cells by alternative splicing (for VEGF-A, VEGF-B, and PGF) and processing (VEGF-A, VEGF-C, and VEGF-D). VEGF gene expression is physiologically regulated by oxygen tension—in hypoxia condition, the transcription factor HIF-1 (hypoxia-inducible transcription factor 1) binds to the hypoxia-responsive enhancer elements (HREs) at VEGF gene affecting transcriptional upregulation [31,32,33]. Moreover, some growth factors and cytokines, including tumor growth factor (TGF), basic fibroblast growth factor (FGF-2), interleukin-1 and interleukin-6 (IL-1, IL-6) can act synergistically with hypoxia [34]. VEGF made up of 121 and 165 amino acids and is produced mainly by neutrophils, platelets, endothelial cells, fibroblasts, epithelial cells, and macrophages in a soluble and freely diffusible form, whereas VEGF consisted of 189 and 206 amino acids is associated with cells’ surface. VEGF acts biologically by receptors tyrosine kinases (RTKs). These receptors have three parts: an extracellular immunoglobulins-like domain, a middle part located in the thickness of the cell membrane, and an intra-cytoplasmic part. VEGF binds to trans-membrane tyrosine kinase receptors, inducing their dimerization and transphosphorylation. VEGF-A binds to VEGFR2 (also called KDR/Flk-1) and VEGFR1 (Flt-1), VEGF-C and VEGF-D bind VEGFR2 and VEGFR3 (Flt4), PlGF and VEGF-B bind only to VEGFR1, and VEGF-E binds only to VEGFR2. VEGFRs differ considerably in signaling properties and are expressed by endothelial cells, epithelial cells, or activated macrophages. In addition, it was found that Neuropilin-1, a trans-membrane protein lacking tyrosine kinase activity, acts as a co-receptor for VEGF-A [35,36]. VEGF plays a key role in the process of vasculogenesis (the de novo formation of the embryonic circulatory system) and angiogenesis (the growth of blood vessels from pre-existing vasculature) in physiological conditions, i.e., during post-natal and skeletal growth, reproductive functions, embryogenesis with endothelial cells’ growth, menstrual cycle, and wound healing. On the other hand, VEGF has also been implicated in pathological angiogenesis, e.g., in cancers and metastasis, retinal neovascularization during DR, age-related macular degeneration, chronic obstructive pulmonary disease, asthma, ischemic heart disease, and rheumatoid or autoimmune diseases [37,38]. Drugs such as aflibercept (binds to circulating VEGF and acts like a “VEGF trap”), bevacizumab and ranibizumab (recombinant humanized monoclonal antibodies that blocks angiogenesis by inhibiting VEGF), or pegaptanib (a pegylated anti-VEGF aptamer, a single strand of nucleic acid that binds to the 165 isoform of VEGF) can inhibit VEGF and control or slow some of those diseases [39,40].

## 3. Therapeutic Concepts in Diabetic Retinopathy

### 3.1. Prevention

Metabolic control should be used clinically to inhibit the development or progression of DR. The most important factor in minimizing the onset of DR is control of hyperglycemia—trials that involved patients both with type 1 diabetes and with type 2 diabetes, showed that tight control of glycated hemoglobin levels and intensive glycemic control may lead to even a 20% reduction in risk of DR [41,42,43]. Moreover, intensive blood pressure control is also beneficial in lowering risk and progression of DR. The next step to decrease progression of DR is treatment of dyslipidemia often connected with DM and hypertension as a metabolic syndrome. Patients with type 2 diabetes should receive statin and/or fenofibrate to get a significant reduction in risk and progression of DR. Additionally, early detection underpins the importance of DR screening and surveillance—for this reason people with diabetes are offered annual screening for the presence of retinopathy. All diabetic screening programs require digital fundus photographs to be taken [44].

### 3.2. Specific Therapeutic Options

In addition to optimal medical control of blood pressure and serum cholesterol and glucose level, several intraocular managements have become standard treatments for DR. Ophthalmological treatment by inhibiting the inflammatory pathway reduces vascular permeability, contributes to the breakdown of the BRB, inhibits leukostasis, inhibits VEGF gene transcription and translation and finally, reduces the risk of visual loss in eyes with sight-threatening complications. Inflammation and VEGF-mediated pathways are crucial in the development and progression of diabetic retinopathy and diabetic macular edema (DME). According to mechanisms, the ocular therapy for diabetic retinopathy and maculopathy includes anti-VEGF drugs, corticosteroids, and laser treatment.

#### 3.2.1. DME Treatment

The exact mechanism of laser photocoagulation in DME treatment is still unknown. It is suggested that it combines the occlusion of leaking vessels, especially microaneurysms and the destruction of ischemic parts of retina. It improves oxygenation to areas located near the treatment ones and contributes to the reduction of proangiogenic factors and cytokines release. The complications after laser treatment include visual field sensitivity deterioration, impairment of color, night vision and sensitivity, enlargement of laser scar, secondary neovascularization, and subretinal fibrosis. The paradigms for treatment of DME have been changing during last years. Intravitreal injections of anti-VEGF have almost expelled the laser treatment for DME.

According to the Guidelines for the Management of Diabetic Macular Edema by the European Society of Retina Specialists (EURETINA) [45] relative indications for using conventional laser therapy are the vasogenic subform of DME with focally grouped microaneurysms and leaking capillaries, central retinal thickness less than 300 µm, and eyes with persisted vitreomacular adhesions. Nowadays, more and more often, we use a subthreshold grid retinal laser which minimizes the destructive consequences of conventional laser therapy. It is achieved by reducing the duration of light exposure and focusing the treatment area only to the retinal pigment epithelium (RPE). Few clinical trials have suggested that subthreshold grid laser is as effective as conventional therapy, but more time is needed for proving that [46,47]. According to EURETINA, early diffuse edema is suggested as a cheaper method than intravitreal injections. The first line therapy in diabetic macular edema (DME) is anti-VEGF intravitreal injection.

We have a few medicaments that we use in treatment of DME: bevacizumab, ranibizumab, aflibercept and the newest one, brolucizumab. The DRCR.net trial compared three of them—bevacizumab, ranibizumab, and aflibercept, and recommends choosing the drug depending on best corrected visual acuity (BCVA) [48]. In this trial patients were assessed with a letter score, which is based on number of letters that patient can read from the ETDRS chart. There are also different types of testing distance visual acuity like Snellen or LogMAR notation. It is always crucial to indicate which notation is used, as lower value in Snellen notation means lower acuity contrary to LogMAR, in which it is better acuity [49].

Using Table 3 it is easy to convert each score:

According to the DRCR.net trial results, aflibercept and ranibizumab are more sufficient for the group with BCVA letter score less than 69 (20/50 or worse in Snellen equivalent). For patients with BCVA 69 or more letters, bevacizumab is the only one used off-label, but the cost is much less than the others. Aflibercept is the drug of choice for DME patients with BCVA below 69 letters as more sufficient than bevacizumab in two years and ranibizumab in first year of treatment. However, not all patients respond properly with this type of treatment. About 40% patients have persisted edema after anti-VEGF monthly injections [50]. For this group, corticosteroid therapy may play a significant role. What is worth noticing for this group is the various mechanisms which lead to an anti-inflammatory effect with the decrease of inflammatory mediators and VEGF. Because of that, they deal with more targets of DME mechanisms than anti-VEGF drugs. These mechanisms can be divided into three groups: anti-inflammatory, vascular and anti-edematous effect. The vascular effect consists of blocking the VEGF-mediated blood-ocular barrier breakdown and edema [51]. Apart from that, corticosteroids inhibit the expression of pathologic mediators such as MCP1, TNF, IL1b, IL6, ICAM1, and SDF-1, which play a role in the breakdown of this barrier [52]. This group of medicaments also stabilizes vessels [53]. The anti-edematous effect is achieved by diminishing edema of Müller cells and restoring potassium and water homeostasis [53,54,55]. We can choose between three drugs available commercially: triamcinolone acetonide, dexamethasone implant (Ozurdex) and fluocinolone (Iluvien). In case of treatment with corticosteroids, cataract development and rise of intraocular pressure should be considered. Corticosteroids have been shown effective in treatment of naive eye and those that do not respond to anti-VEGF treatment. The latest study shown that while making decision for treatment for an individual patient, we should assess probable biomarkers of inflammation in our diagnostic process [56,57,58,59]. The first one is hyperreflective retinal foci (HRF), which can represent hard exudates, degenerated photoreceptors or macrophages engulfing them, anteriorly migrated RPE cells, retinal vessels or activated microglial cells [60]. Vujosevic et al. [61] has shown that HRF can be distinguished in patients with early diabetic retinopathy without DME and patients with diabetes but without diabetic retinopathy, mainly in the inner parts of the retina. They suggested that these HRF are marks of the inflammation process, and they appear as a response to early microglia activation. The next one is subretinal fluid [62]. It can occur in about 15–30% patients with DME. It is more frequent with thicker choroid and with occurrence of hyperreflectivity foci. The other markers worth mentioning are the disorganization of the inner retinal layers (DRIL) and its extension and choroidal vascularity index (CVI) [63]. Greater reduction of DRIL extension has been observed in patients treated with steroids. CVI is the ratio between choroidal stroma and the vessels which may be useful in assessment of choroidal congestion [64]. Arrigo et al. reported that high amount of choroidal HRF and low CVI values seems to be response to chorioretinal inflammation. According to Udaondo et al. [56], the best candidate for good response with corticosteroids is with subretinal fluid, intraretinal inflammatory cysts, and HRF.

#### 3.2.2. Proliferative Diabetic Retinopathy (PDR) Treatment

Three large clinical trials were undertaken: the British multicenter trial using xenon arc photocoagulation [65] and the National Eye Institute’s Diabethic Retinopathy Study (DRS) [66], in which xenon arc and argon laser photocoagulation were compared to no photocoagulations in PDR and Early Treatment Diabetic Retinopathy Study (ETDRS) [67]. The DRS and ETDRS have proved that panretinal photocoagulation (PRP) significantly lowers risk of severe vision loss from PDR. The mechanism of PRP is not fully known. It is supposed that laser treatment of ischemic areas makes the retina produce less neovascularization-inducing growth factors (e.g., VEGF). The first effect after laser treatment is more blood flow from the choroid to the inner retina [68]. We must bear in mind that PRP has also complications such as loss of visual function, damage to posterior ocular structures, macular edema, choroidal detachment, secondary angle closure, iritis and increased ocular pressure, and exudative retinal detachment. It has been reported that using anti-VEGF intravitreal injections may diminish ischemia-related ocular neovascularization. Avery et al. [69] have shown the effect of bevacizumab 24 h after injection with a duration of study of 2–11 weeks. Results of DRCR.net study confirmed that patients treated with anti-VEGF had less visual acuity loss, less visual field loss, less need for vitrectomy, and less DME development [70]. These results may be vital while making a decision for treatment for PDR eyes, especially in young patients.

## 4. Conclusions

Diabetic retinopathy is a leading cause of vision loss in working-age patients in highly developed countries and is a serious cause of blindness in the world. The population of patients touched by this problem systematically grows. We are facing the problem of accurate care for this patient, an early and easy diagnostic process, and better outcomes. Numerous studies have supported the hypothesis that diabetic retinopathy is a neurovascular disease with neurodegeneration as an early event in the diabetic retina. The molecular mechanism of neuronal damage in patients with DR relates to excitotoxic metabolites, altered neurotrophic support/signaling and oxidative stress. Undoubtedly, hyperglycemia causes abnormalities of biochemical pathways and an additionally dysregulated level of VEGF to promote the development of inflammation and retinal hypoxia. Considering that DR is a part of systemic diabetes progression, controlling of primary disease should be a necessary background of DR prevention and treatment. Novel therapeutic strategies, like anti-VEGF or steroid option, concentrate on a more fundamental level of DR pathophysiology, and because of that, improve visual acuity and patients’ quality of life. The intravitreal injections have almost expelled laser treatment and given a significant improvement in anatomical and functional results. We must be aware that the response for treatment is not equal for each patient because the contribution of mechanisms is different and different types of care are required. Hopefully, by using multimodal imaging and assessment of those markers mentioned above, we will be able to tailor more individualized paths for each patient.

## Figures and Tables

**Table 1:** Classification of diabetic retinopathy—according with [18].

Type	Ophthalmoscopic Features
Mild NPDR	Microaneurysms
Moderate NPDR	At least two of the following features: * Microaneurysms * Retinal hemorrhages * Hard exudates
Severe NPDR	Any one of the following features: * 20 hemorrhages in each of the four quadrants * Venous beading in two quadrants * IrMAs in one quadrant
PDR	At least one of the following features: * Neovascularization * Vitreous hemorrhage

NPDR—non-proliferative diabetic retinopathy; PDR—proliferative diabetic retinopathy; IrMAs—Intraretinal microvascular abnormalities.

**Table 2:** The VEGF-family.

Member of the VEGF-Family	Chromosomal Location	Receptor	Function
VEGF-A	6p23.1	VEGFR1, VEGFR2, NRP1	* angiogenesis: mitosis and migration of endothelial cells, and astrocytes * creation of blood vessel lumen and fenestrations * chemotactic factor for granulocytes and macrophages * vasodilation (indirectly by NO release)
VEGF-B	11q13	VEGFR1, NRP1	* embryonic angiogenesis * protective for neurons in the retina and the cerebral cortex during stroke * protective for motoneurons during motor neuron diseases such as amyotrophic lateral sclerosis
VEGF-C	4q34	VEGFR2, VEGFR3, NRP2	* promote the growth of lymphatic vessels (lymphangiogenesis)
VEGF-D	Xp22.31	VEGFR2, VEGFR3	* similar to those of VEGF-C * necessary for the development of lymphatic vasculature surrounding lung bronchioles
PlGF	14q24	VEGFR, NRP1, NRP2	* vasculogenesis * angiogenesis during ischemia, inflammation, wound healing, and cancer * regulates growth and differentiation of trophoblasts in the placenta during pregnancy

VEGF—vascular endothelial growth factor; VEGFR—vascular endothelial growth factor receptor; NRP—neuropilin; PlGF—placenta growth factor; NO—nitric oxide.

**Table 3:** Visual Acuity convertion chart.

Letter Score	LogMAR Value	Snellen Equivalent
5	1.6	20/800
10	1.5	20/640
15	1.4	20/500
20	1.3	20/400
25	1.2	20/320
30	1.1	20/250
35	1.0	20/200
40	0.9	20/160
45	0.8	20/125
50	0.7	20/100
55	0.6	20/80
60	0.5	20/63
65	0.4	20/50
70	0.3	20/40
75	0.2	20/32
80	0.1	20/25
85	0.0	20/20
90	−0.1	20/15
95	−0.2	20/12

## Data Availability

Not applicable.
